# Spinosin Attenuates HCl/Ethanol-Induced Gastric Mucosal Injury by Modulating Oxidative Stress, Inflammation, Apoptosis, and Nrf2/HO-1-Associated Responses

**DOI:** 10.3390/biomedicines14071452

**Published:** 2026-06-26

**Authors:** Selcan Cesur, Berrin Yalinbas-Kaya

**Affiliations:** Department of Gastroenterology, Ministry of Health Eskisehir City Hospital, 26080 Eskisehir, Türkiye; yalinbaskayaberrin@gmail.com

**Keywords:** spinosin, gastric ulcer, anti-inflammatory effect, Nrf2/HO-1, apoptosis

## Abstract

**Background/Objectives:** Spinosin, a flavone glycoside derived from medicinal plants, has been widely studied for its neuroactive properties; however, its effects on gastric injury remain unclear. Therefore, this study investigated the potential protective role of spinosin against HCl/ethanol-induced gastric lesions and its association with Nrf2/HO-1-related antioxidant responses. **Methods:** Gastric ulceration was experimentally induced in male Swiss albino mice by intragastric administration of 0.3 M HCl in 70% ethanol. Spinosin was administered orally at doses of 10 and 20 mg/kg, while omeprazole (20 mg/kg) was used as a reference treatment. **Results:** HCl/ethanol exposure led to pronounced oxidative stress and inflammatory responses, as reflected by increased levels of MDA, NFκB, IL-6, TNF-α, Cox-2, iNOS, IL-1β, Bax, and Cas-3, along with reduced antioxidant enzyme activities (GSH, SOD, CAT), decreased PGE2 and NO levels, and downregulation of Nrf2, HO-1, and Bcl-2 expression. Spinosin administration significantly attenuated gastric injury, suppressed pro-inflammatory mediators, reduced markers of lipid peroxidation and apoptosis, and enhanced antioxidant defenses. In parallel, spinosin treatment was associated with increased expression of Nrf2, HO-1, and Bcl-2. **Conclusions:** These findings suggest that spinosin mitigates gastric damage and is associated with attenuation of oxidative stress, inflammatory responses, and apoptosis, suggesting a possible contribution of Nrf2/HO-1-related antioxidant responses.

## 1. Introduction

Gastric ulceration is a disorder characterized by injury to the gastric mucosa and remains one of the most common gastrointestinal diseases worldwide [1]. In addition, gastric ulcers affect more than 10% of the global population, and approximately one-tenth of these cases carry a potential risk of progression to malignancy [2]. Key contributors to the development of this condition include excessive alcohol consumption, poor dietary habits, tobacco use, psychological stress, and the inappropriate or uncontrolled use of non-steroidal anti-inflammatory drugs (NSAIDs) [3]. The ulcerogenic effects of these risk factors are primarily consistent with enhanced oxidative damage and inflammation, supporting the use of antioxidant- and anti-inflammatory-based interventions for the prevention and management of gastric ulcers [4]. Moreover, gastric mucosal injury arising from oxidative stress and inflammatory reactions becomes increasingly severe under the continuous influence of gastric acid [5]. Accordingly, the HCl/ethanol-induced gastric ulcer model employed in this study is extensively utilized to elucidate the pathogenic mechanisms of gastric ulceration and to assess the therapeutic potential of candidate anti-ulcer agents [3].

Nrf2 is a central transcriptional regulator responsible for coordinating antioxidant defense mechanisms that preserve redox balance at the cellular and tissue levels under oxidative challenge [6,7]. Moderate increases in reactive oxygen species (ROS) can activate Nrf2 as an adaptive cytoprotective response, promoting its nuclear translocation and the induction of heme oxygenase-1 (HO-1) [8]. However, excessive or sustained oxidative stress may impair Nrf2/HO-1 signaling and thereby contribute to oxidative tissue injury. Concomitant NFκB activation drives an inflammatory cascade, initiating TNF-α synthesis and secretion and subsequently enhancing the expression of additional pro-inflammatory cytokines, including IL-6 and IL-1β [9]. Activation of the Nrf2/HO-1 signaling axis can suppress inflammation by preventing the nuclear translocation of NFκB and thereby inhibiting NFκB-dependent inflammatory responses [10]. Cyclooxygenase-2 (Cox-2) is functionally linked to the NFκB signaling network and contributes to the pathophysiology of numerous inflammation-driven disorders accompanied by NFκB activity [11]. Accordingly, a primary therapeutic objective in controlling inflammation is to promote Nrf2 activation while suppressing NFκB signaling, thereby limiting the release of pro-inflammatory cytokines [6].

Given the adverse effects associated with many synthetic anti-ulcer agents, increasing attention has been directed toward natural products and bioactive compounds as alternative strategies for gastrointestinal protection [5]. Numerous plant-derived compounds have demonstrated gastroprotective effects through antioxidant, anti-inflammatory, antisecretory, and cytoprotective mechanisms [12]. However, despite the growing body of evidence supporting the therapeutic potential of natural products in gastric injury, the gastroprotective activity of spinosin has not yet been investigated.

Spinosin is a plant-derived flavone C-glycoside that has been identified in multiple botanical sources, particularly *Ziziphus jujuba* Mill. var. spinosa, which is recognized as a prominent reservoir of this compound [13], as well as *Passiflora edulis* Sims [14], *Cayaponia tayuya* (Vell.) Cogn [15] and *Leonurus japonicus* Houttn [16]. Previous studies have reported diverse pharmacological properties of spinosin, including neuroprotective, sedative, cognition-enhancing, anti-melanogenic, and potent antioxidant activities [17,18,19,20]. Among these properties, its strong antioxidant potential is of particular interest because oxidative stress plays a pivotal role in the pathogenesis of gastric mucosal injury. Therefore, spinosin may offer gastroprotective benefits through mechanisms associated with the attenuation of oxidative damage and preservation of mucosal integrity.

To the best of our knowledge, no previous study has investigated the gastroprotective potential of spinosin in experimental gastric injury models. Accordingly, the present study was designed to investigate the gastroprotective potential of spinosin using biochemical, oxidative stress, and histopathological assessments.

## 2. Materials and Methods

### 2.1. Chemicals

Spinosin (CAS No. 72063-39-9), ethanol (CAS No. 64-17-5), DMSO (CAS No. 67-68-5), and HCl (CAS No. 7647-01-0) were purchased from Sigma-Aldrich (St. Louis, MO, USA). Omeprazole (CAS No. 73590-58-6) used in the study was obtained from Terra Pharmaceutical Industry (Istanbul, Türkiye). Unless otherwise specified, all reagents were of analytical quality and obtained from reputable commercial sources.

### 2.2. Experimental Design

A total of 49 male Swiss albino mice (4–5 months of age, weighing 30–40 g) were included in the experimental procedures. Animals were sourced from the Experimental Animal Center of Afyon Kocatepe University. The study protocol received ethical clearance from the Local Ethics Committee (26 October 2025; 49533702/343) and followed internationally accepted standards for animal experimentation. Mice were kept in a controlled environment (22 ± 2 °C, 55–60% humidity) under a 12 h light/dark cycle, with unrestricted access to standard feed and water. To prepare for ulcer induction, animals were fasted for 24 h.

A total of seven groups were established, each containing seven mice. The experimental doses of HCl/ethanol, spinosin, and omeprazole were adopted from previously published protocols [18,19,21,22], and group details are provided in Table 1. The doses of spinosin (10 and 20 mg/kg) were selected based on previous studies demonstrating antioxidant, anti-inflammatory, and tissue-protective effects at these dose levels without evidence of toxicity in mice. In addition, the use of two dose levels enabled a preliminary assessment of dose responsiveness. Treatments (spinosin and omeprazole) were dissolved in 0.1% DMSO and delivered orally for three days. Ulcer formation was triggered by the administration of HCl/ethanol one hour after the final dose.

After a 4 h interval, anesthesia was performed using ketamine (80 mg/kg; i.p.) and xylazine (10 mg/kg; i.p.). Blood and stomach tissues were collected using intracardiac puncture. Serum was obtained following centrifugation at 600× *g* for 15 min. Gastric samples were homogenized in a buffer (Tris–HCl; 0.15 M, pH 7.4) and centrifuged at 2500× *g* (10 min). All collected samples were stored at −80 °C.

### 2.3. Determination of Gastric pH and Gastric Wall Mucus

Under anesthesia, the pylorus was carefully ligated prior to euthanasia to allow collection of gastric secretions. Following euthanasia, the stomachs were excised, and the accumulated gastric contents were collected and centrifuged (4000 rpm, 15 min). The pH of the gastric juice was determined using a digital pH meter (Sartorius, Göttingen, Germany) [23].

To assess gastric mucus levels, a defined section of the stomach was placed in an Alcian blue solution (0.02%, 10 mL) prepared in a sucrose (0.16 M)–sodium acetate (0.05 M) buffer adjusted to pH 5.8 and maintained at 25 °C for 24 h to allow dye binding. Following incubation, the samples were centrifuged (3000 rpm—10 min—4 °C). The supernatant absorbance was recorded at 620 nm, and gastric mucus content was expressed as mg per g of tissue and calculated according to the extent of Alcian blue binding, with concentrations determined from a standard calibration curve generated using known Alcian blue solutions [24].

### 2.4. Macroscopic Examination of Gastric Tissue and Ulcer Lesions

Following removal, the stomachs were incised longitudinally along the greater curvature, gently washed with isotonic saline, and subjected to gross morphological inspection. The extent of gastric injury was assessed independently by observers blinded to the experimental groups. Lesions were graded according to a standardized scoring system: 0 (absence of lesions), 1–2 (minor mucosal damage), 3–4 (localized ulcerative lesions), 5–6 (pronounced ulcer formation), and 7 (severe and widespread ulceration) [25]. In addition, photographic documentation of the gastric mucosa was obtained, and ulcerated regions were quantitatively evaluated using digital image analysis software (ImageJ, version 1.53r; NIH, USA). The total damaged mucosal surface was calculated and expressed in square millimeters [26].

### 2.5. Biochemical Analyses

Malondialdehyde (MDA) levels, as an indicator of lipid peroxidation, were quantified in gastric tissue homogenates using the thiobarbituric acid-reactive substances (TBARS) assay according to Ohkawa et al. [27]. Reduced glutathione (GSH) content was determined in gastric tissue homogenates following the method described by Draper and Hadley [28]. The activities of antioxidant enzymes, superoxide dismutase (SOD) and catalase (CAT), were measured spectrophotometrically using standard methods [29,30]. Protein concentrations were determined by the Lowry method [31] and biochemical parameters were normalized to protein content where appropriate. All spectrophotometric measurements were performed using a Shimadzu UV–Vis spectrophotometer (Shimadzu, Tokyo, Japan). Levels of IL-6, PGE2, and nitric oxide (NO) were measured in gastric tissue homogenate supernatants using commercially available ELISA kits (Sunlong Biotech, Hangzhou, Zhejiang, China; catalogue numbers: SL0326Mo, SL0461Mo, and SL0615Mo, respectively) according to the manufacturer’s instructions. Absorbance values were recorded at 450 nm using a microplate reader. Results were expressed in the units specified by the respective assay kits.

### 2.6. Relative mRNA Expression Levels of the Target Genes

RNA isolation from gastric tissues was carried out using the A.B.T.™ Blood/Tissue RNA Purification Kit. The purified RNA was reverse transcribed to generate cDNA templates. Quantification of transcripts related to inflammatory, apoptotic, and antioxidant pathways (NFκB, Cox-2, iNOS, IL-1β, Bax, TNF-α, HO-1, Cas-3, Nrf2, and Bcl-2) was performed using real-time PCR. Custom oligonucleotide primers were taken from Sentegen Biotec (Ankara, Türkiye), and detailed sequences are presented in Table 2. Amplification reactions were performed in triplicate. Relative gene expression was calculated using the comparative Ct (2^−ΔΔCt^) method following normalization to β-actin [22].

### 2.7. Histopathological Analysis

Gastric samples were fixed in 10% neutral-buffered formalin, embedded in paraffin, and sectioned into 5 µm thick slices using a microtome (Leica, Nussloch, Germany) [7]. Tissue sections were stained with hematoxylin and eosin (H&E) and examined under a light microscope equipped with a digital imaging system (Nikon DS-Fi3, Tokyo, Japan). Histopathological evaluation focused on desquamation and shedding of the mucosal epithelium, submucosal edema, submucosal mononuclear cell infiltration, lymphatic vessel dilation, and vascular hyperemia. The severity of each lesion was assessed using a semi-quantitative scoring system ranging from 0 to 3 (0 = absent, 1 = mild, 2 = moderate, and 3 = severe). Histopathological evaluation was performed by a pathologist blinded to the treatment groups. Histopathological scores were subsequently subjected to statistical analysis as ordinal variables.

### 2.8. Immunohistochemical Analysis

Paraffin sections were deparaffinized, rehydrated, and cleared, followed by inhibition of endogenous peroxidase activity using 1% H_2_O_2_. Antigen retrieval was performed by heat treatment in citrate buffer. After washing, sections were incubated with primary antibodies (Table 3) overnight at 4 °C. Subsequently, HRP-conjugated secondary antibody was applied, and immunoreactivity was visualized using DAB. Sections were counterstained with hematoxylin and examined under a light microscope (200×). Five randomly selected non-overlapping fields from each section were photographed under 200× magnification. The percentage of positively stained area was quantified using ImageJ software. The investigator performing image analysis was blinded to the experimental groups. The mean value obtained from five fields was used for statistical analysis.

### 2.9. Statistical Analysis

Statistical analyses were performed using GraphPad Prism 8.0 (GraphPad Software, San Diego, CA, USA). The normality of continuous variables was assessed using the Shapiro–Wilk test. Data showing a normal distribution were analyzed using one-way analysis of variance (ANOVA), followed by Tukey’s post hoc test, and are presented as mean ± standard deviation (SD). Histopathological scoring data, which are ordinal in nature, were expressed as median (minimum–maximum) and analyzed using the Kruskal–Wallis test followed by Dunn’s multiple comparisons test. Differences were considered statistically significant at *p* < 0.05.

## 3. Results

### 3.1. Restorative Effect of Spinosin Against HCl/Ethanol-Induced Gastric Injury

Macroscopic examination revealed no visible gastric alterations in the control, DMSO, and spinosin (20 mg/kg)–treated groups (Figure 1A,B,D). In contrast, mice exposed to HCl/ethanol exhibited extensive focal hemorrhagic ulcerative lesions throughout the gastric mucosa, characterized by hemorrhagic necrosis and loss of epithelial integrity (Figure 1C). Co-administration of spinosin at doses of 10 and 20 mg/kg markedly attenuated ulcer formation compared with the HCl/ethanol group (*p* < 0.0001; Figure 1E,F). Similarly, treatment with omeprazole, used as a positive control, significantly reduced HCl/ethanol-induced gastric damage (Figure 1G). Quantitative analysis demonstrated a 74.15% increase in gastric mucosal lesion area in the HCl/ethanol group relative to the normal control group (*p* < 0.0001). Conversely, oral administration of spinosin at 10 and 20 mg/kg, as well as omeprazole, reduced mucosal injury by 10.25%, 27.93%, and 46.57%, respectively, when compared with the HCl/ethanol-treated group (*p* < 0.0001; Figure 1H).

Assessment of gastric mucus content showed a significant depletion in the HCl/ethanol group compared with the normal control group. However, mice treated with spinosin at doses of 10 and 20 mg/kg displayed significantly higher mucus levels than those in the HCl/ethanol group (*p* < 0.0001). Specifically, gastric mucus content in the HCl/ethanol group decreased by 73.35% relative to controls. In contrast, treatment with spinosin at 10 and 20 mg/kg and omeprazole resulted in increases of 14.31% (*p* < 0.0075), 21.14% (*p* < 0.0001), and 50.77% (*p* < 0.0001), respectively, compared with the HCl/ethanol group (Figure 2A). No statistically significant differences in mucus content were observed between the control group and the DMSO or spinosin (20 mg/kg)–only groups. Additionally, gastric pH values were significantly reduced following HCl/ethanol administration compared with control (*p* < 0.0001). Pretreatment with spinosin at 10 mg/kg (*p* < 0.0004), 20 mg/kg, or omeprazole (20 mg/kg) (*p* < 0.0001) significantly restored gastric pH levels relative to the HCl/ethanol group (Figure 2B). Collectively, these findings indicate that spinosin effectively ameliorates HCl/ethanol-induced reductions in gastric pH and mucus content.

Histopathological evaluation (Figure 3) of hematoxylin- and eosin-stained gastric sections revealed preserved mucosal architecture with no detectable abnormalities in the control, DMSO, and spinosin-treated (20 mg/kg) groups. In contrast, pronounced pathological alterations were observed following HCl/ethanol exposure, including epithelial desquamation and erosion, edema in the submucosal and serosal layers, mononuclear cell infiltration, lymphatic dilation, and vascular hyperemia (*p* < 0.001; Table 4). Treatment with spinosin at doses of 10 mg/kg and 20 mg/kg significantly mitigated these microscopic lesions compared with the HCl/ethanol group (*p* < 0.001). Likewise, omeprazole treatment markedly alleviated histopathological damage, resulting in substantially reduced mucosal injury relative to the HCl/ethanol group (*p* < 0.001).

### 3.2. Restorative Effects of Spinosin on Oxidative Stress–Associated Biochemical Parameters in Gastric Tissue of HCl/Ethanol-Challenged Mice

Because HCl/ethanol-induced gastric injury is closely associated with the development of oxidative stress [22], levels of MDA and GSH, together with the activities of SOD and CAT, were assessed in gastric homogenates. Gastric MDA concentrations (Figure 4A) were markedly elevated following HCl/ethanol administration; however, pretreatment with spinosin at doses of 10 and 20 mg/kg, as well as omeprazole (20 mg/kg), significantly attenuated this increase (*p* < 0.0001). In contrast, HCl/ethanol exposure resulted in a pronounced depletion of gastric GSH levels, which were significantly restored by pretreatment with spinosin at 10 mg/kg (*p* < 0.0002), spinosin at 20 mg/kg (*p* < 0.0001), and omeprazole at 20 mg/kg (*p* < 0.0001) (Figure 4B). A similar pattern was observed for antioxidant enzyme activities, as both SOD (Figure 4C) and CAT (Figure 4D) activities, which were suppressed by HCl/ethanol challenge, were significantly enhanced following administration of spinosin or omeprazole (*p* < 0.0002–0.0001). These findings indicate that spinosin exerts a protective effect against HCl/ethanol-induced gastric damage by mitigating oxidative stress through its antioxidant properties. Moreover, no significant alterations in these biochemical parameters were detected in the DMSO-treated or spinosin-only (20 mg/kg) groups when compared with the control group.

### 3.3. Regulatory Effects of Spinosin on Inflammation-Associated Mediators in the Serum and Gastric Tissue of HCl/Ethanol-Treated Mice

IL-6 and the gastric mucosal mediators PGE2 and NO play important roles in gastric mucosal homeostasis and inflammatory responses. In addition, NFκB, TNF-α, iNOS, Cox-2, and IL-1β are closely associated with gastric inflammation. Exposure to HCl/ethanol resulted in a marked increase in IL-6 concentrations in both serum and gastric tissues relative to controls (Figure 5A,B; *p* < 0.0001). Conversely, levels of PGE2 (Figure 5C,D) and NO (Figure 5E,F) were significantly reduced (*p* < 0.0001). In addition, expression of NFκB, TNF-α, iNOS, Cox-2, and IL-1β (Figure 6A–E) was strongly elevated at the mRNA level, accompanied by increased immunoreactivity, as observed by immunohistochemistry (Figure 7; *p* < 0.0001).

Administration of spinosin at 10 and 20 mg/kg, along with omeprazole at 20 mg/kg, effectively counteracted these changes by normalizing inflammatory mediator levels and reducing the overexpression of related genes. No significant differences were observed in animals treated with DMSO or spinosin alone compared with the control group.

### 3.4. Modulatory Role of Spinosin on Nrf2/HO-1 Signaling and Apoptosis-Related Markers in Gastric Tissue of HCl/Ethanol-Exposed Mice

The Nrf2/HO-1 signaling system is central to maintaining redox balance during oxidative challenge. In the present model, HCl/ethanol administration resulted in a marked decline in both gene expression (Figure 8A,B; *p* < 0.0001) and immunohistochemical detection (Figure 9; *p* < 0.0001) of Nrf2 and HO-1 in gastric tissues. These suppressive effects were significantly attenuated by spinosin treatment (10 and 20 mg/kg) and omeprazole (20 mg/kg), which restored the expression of these antioxidant-related factors. Neither DMSO nor spinosin alone produced any detectable effect on Nrf2 or HO-1 expression.

In terms of apoptosis-related parameters, exposure to HCl/ethanol induced a pronounced upregulation of Cas-3 and Bax, along with a reduction in Bcl-2 expression at both transcriptional and protein levels (Figure 8C–E and Figure 9; *p* < 0.0001). Administration of spinosin and omeprazole significantly counteracted these effects, lowering Cas-3 and Bax levels while increasing Bcl-2 expression. No meaningful changes were observed in the DMSO or spinosin-alone groups relative to controls.

## 4. Discussion

Numerous studies have highlighted alcohol consumption as a major contributing factor in the development of gastric injury, particularly in the progression of ulceration and hemorrhagic lesions [32,33,34]. The HCl/ethanol-induced gastric injury model is widely employed to investigate the mechanisms underlying ulcer development and to evaluate the protective efficacy of pharmacological agents or naturally derived compounds [22,34]. Accordingly, the present study aimed to investigate the gastroprotective efficacy of spinosin, a compound with well-established pharmacological properties, in the context of gastric ulceration. Notably, spinosin markedly reduced the ulcer index and pathological damage induced by HCl/ethanol, thereby enhancing ulcer inhibition. Ultimately, these protective effects were associated with attenuation of oxidative stress, modulation of inflammatory biomarkers, and restoration of Nrf2/HO-1-related antioxidant responses.

HCl/ethanol exposure promotes gastric mucosal ulceration and hemorrhage by suppressing mucus secretion while simultaneously intensifying gastric acidity [4]. Consistent with this mechanism, the HCl/ethanol-treated group in the present study exhibited pronounced hemorrhage and extensive ulcerative lesions, in agreement with observations reported in previous investigations [22,23]. Treatment with spinosin at 10 and 20 mg/kg produced a significant reduction in gastric lesion area and was accompanied by elevations in gastric pH and mucus content. These protective effects were further corroborated by histopathological findings, including a marked reduction in submucosal edema and inflammatory cell infiltration. Collectively, these results are consistent with previous reports evaluating the gastroprotective potential of various natural compounds with established antioxidant activity, including spinosin [35], and align well with findings from related experimental studies [23,33,34].

Ethanol-induced gastric injury is characterized by an excessive accumulation of free radicals and lipid peroxidation by-products such as MDA within gastric tissue, while concurrently leading to a marked depletion of antioxidant defenses, including GSH levels and the activities of SOD and CAT [3,36]. In line with these observations, our findings demonstrated that HCl/ethanol exposure led to a significant elevation in MDA levels, accompanied by marked reductions in GSH content as well as SOD and CAT activities in gastric tissue. In contrast, administration of spinosin (10 and 20 mg/kg), similar to omeprazole treatment, effectively counteracted HCl/ethanol–induced lipid peroxidation and restored the suppressed antioxidant defense system. These outcomes suggest that spinosin may contribute to the attenuation of gastric injury through its antioxidant properties by enhancing endogenous antioxidant defenses and limiting oxidative damage [20,37,38].

Maintenance of gastric tissue homeostasis and effective ulcer repair largely depend on the protective actions of PGE2 and NO [39]. NO contributes to gastric protection by sustaining mucosal blood flow and stimulating the secretion of mucus and bicarbonate, while PGE2 plays a complementary role by regulating gastric acid and cytotoxic mediator release, stabilizing mast cell membranes, and promoting tissue repair processes, collectively enhancing resistance to ulcer formation [23]. Ethanol exposure further amplifies gastric inflammation by stimulating macrophage-driven release of pro-inflammatory mediators such as IL-6 [40]. The present findings demonstrated that HCl/ethanol exposure resulted in a marked reduction in gastric PGE2 and NO levels, concomitant with a significant elevation in IL-6 concentrations. In contrast, treatment with omeprazole and spinosin effectively modulated PGE2, NO, and IL-6 levels in both serum and gastric homogenates when compared with the HCl/ethanol-treated group. Supporting these observations, previous studies have reported that administration of Semen *Ziziphi Spinosae*, the plant source of spinosin, at a dose of 2500 mg/kg significantly attenuated stress-induced gastric ulceration, suggesting that this plant possesses notable anti-ulcerative properties [41]. Although iNOS expression was elevated following HCl/ethanol administration, total NO levels were reduced. This apparent discrepancy may reflect increased consumption of NO under conditions of severe oxidative stress, leading to peroxynitrite formation and reduced NO bioavailability despite enhanced iNOS expression. Similar observations have been reported in experimental gastric injury models [42,43]. The observed changes in PGE2, NO, and IL-6 were accompanied by improved preservation of gastric tissue integrity in spinosin-treated animals.

Ethanol challenge is known to stimulate immune signaling pathways that result in increased production of pro-inflammatory mediators [22]. In the context of gastric ulcers, NFκB signaling amplifies inflammation through the induction of TNF-α and subsequent upregulation of cytokines, including iNOS, Cox-2, and IL-1β [44]. In the present study, exposure to HCl/ethanol resulted in a pronounced upregulation of gastric NFκB, TNF-α, Cox-2, IL-1β, and iNOS gene expression, accompanied by increased immunoreactivity for these markers in mice. In contrast, administration of spinosin at doses of 10 and 20 mg/kg, as well as omeprazole, markedly suppressed these elevated inflammatory mediators. Supporting these findings, previous in vitro evidence has demonstrated that spinosin attenuates doxorubicin-induced cardiotoxicity in H9c2 cells by downregulating the expression of TNF-α, IL-6, Cox-2, and NFκB [45]. Another study reported that spinosin exhibits a strong binding affinity toward NFκB and its upstream inhibitor κB kinase, and in silico analyses revealed binding energies comparable to those of the reference inhibitor MG-132, with a corresponding suppressive effect on nuclear p65 protein levels [46]. These results suggest that spinosin treatment was associated with reduced expression of NFκB-related inflammatory markers in gastric tissue.

By regulating intrinsic antioxidant defenses, the Nrf2 pathway serves as a central mechanism safeguarding cells from oxidative stress–induced damage [23]. Oxidative challenge disrupts the cytoplasmic Nrf2–Keap1 complex, enabling Nrf2 to trigger antioxidant response element–driven expression of protective enzymes, including HO-1, SOD, and CAT [32]. Consistent with earlier reports [47,48], the present study revealed markedly reduced Nrf2 and HO-1 levels within ulcerated gastric regions following HCl/ethanol exposure. In contrast, treatment with spinosin and omeprazole was associated with increased Nrf2 expression and upregulation of HO-1. These findings suggest that the gastroprotective effects of spinosin are associated with increased Nrf2 and HO-1 expression in gastric tissue. Moreover, the current results align with previous studies demonstrating the regulatory influence of spinosin on the Nrf2/HO-1 pathway [38]. In ethanol-related gastric ulcer formation, oxidative stress triggers mitochondrial apoptotic signaling, marked by Bax activation and downstream Cas-3 engagement, ultimately resulting in cellular apoptosis [23]. In the present study, administration of spinosin markedly increased Bcl-2 expression while significantly suppressing Bax and Cas-3 expression in mice subjected to HCl/ethanol-induced gastric injury. These findings suggest that spinosin treatment was associated with reduced expression of pro-apoptotic markers and increased expression of anti-apoptotic markers in gastric tissue.

Despite the consistent protective effects observed in the present study, certain limitations should be acknowledged. Although spinosin treatment was associated with restoration of Nrf2 and HO-1 expression, the current findings do not establish a direct causal role for the Nrf2/HO-1 pathway in mediating the observed gastroprotective effects. Since pathway-specific inhibition or gene-silencing approaches were not employed, it remains unclear whether activation of Nrf2/HO-1 represents a primary pharmacological action of spinosin or a secondary consequence of reduced oxidative stress and tissue injury. Furthermore, mechanistic interpretation is limited by the absence of complementary protein-based validation methods such as Western blot analysis. Therefore, future studies incorporating loss-of-function strategies and additional molecular approaches are warranted to further clarify the mechanistic basis of spinosin-mediated gastric protection. In addition, epithelial barrier-related proteins such as ZO-1, occludin, and claudins, as well as CYP2E1 expression, were not evaluated in the present study. Future investigations addressing these parameters may provide further mechanistic insight into the gastroprotective effects of spinosin.

## 5. Conclusions

Spinosin markedly mitigated HCl/ethanol-induced gastric mucosal injury and exerted protective effects at macroscopic, histopathological, biochemical, and molecular levels. These effects were associated with restoration of antioxidant defenses, suppression of inflammatory mediators, and modulation of apoptosis-related markers. In addition, spinosin treatment was accompanied by increased expression of Nrf2 and HO-1, suggesting a possible contribution of Nrf2/HO-1-related antioxidant responses to its gastroprotective activity. Although further mechanistic studies are required, the present findings indicate that spinosin may represent a promising natural compound for the prevention or management of gastric mucosal injury.

## Figures and Tables

**Figure 1:**
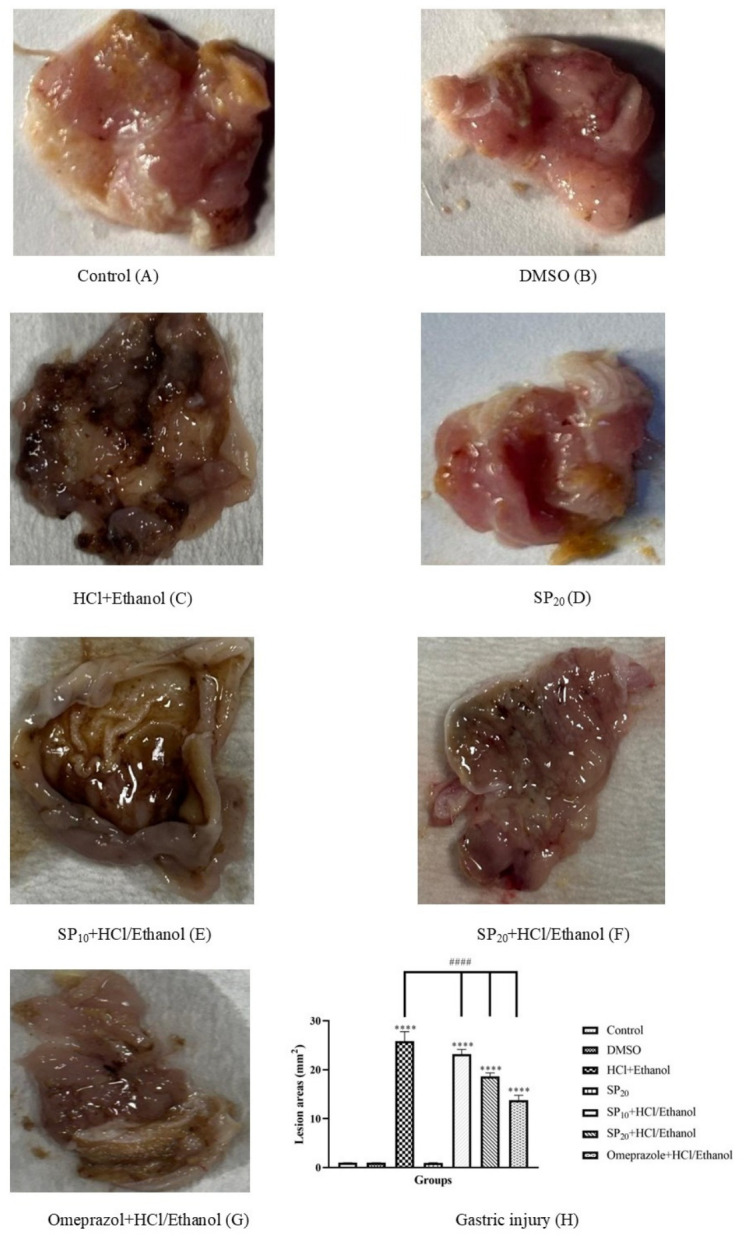
Gross morphological appearance of gastric tissues following acute HCl/ethanol-induced injury. Macroscopic images illustrate gastric specimens obtained from the experimental groups: Control (**A**), DMSO (**B**), HCl/ethanol (**C**), spinosin-treated group (SP_20_) (**D**), SP_10_ pretreatment followed by HCl/ethanol (**E**), SP_20_ pretreatment followed by HCl/ethanol (**F**), and Omeprazole + HCl/ethanol (**G**). Extensive mucosal damage characterized by hemorrhage and surface erosion was observed in the HCl/ethanol group. In contrast, animals pretreated with spinosin exhibited a marked attenuation of gastric lesions, comparable to the protective effect of omeprazole. Panel (**H**) depicts the quantitative measurement of gastric lesion areas (mm^2^). Values are presented as mean ± SD (n = 7 animals per group). **** *p* < 0.0001 compared with the control group; #### *p* < 0.0001 compared with the HCl/ethanol group.

**Figure 2:**
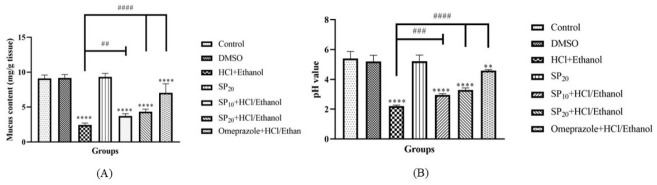
Effects of spinosin on gastric mucus content and intragastric pH in the HCl/ethanol-induced gastric injury model. Panel (**A**) illustrates the alterations in gastric mucus levels (mg/g tissue) across the experimental groups, while Panel (**B**) presents corresponding changes in gastric pH values. Exposure to HCl/ethanol resulted in a marked depletion of gastric mucus and a significant reduction in pH compared with control groups. Pretreatment with spinosin (SP_10_ and SP_20_) significantly restored mucus content and elevated gastric pH. The protective response observed with spinosin was comparable to that achieved with omeprazole treatment. Data are expressed as mean ± SD (n = 7 animals per group). ** *p* < 0.01, **** *p* < 0.0001 versus the control group; ## *p* < 0.01, ### *p* < 0.001, and #### *p* < 0.0001 versus the HCl/ethanol group.

**Figure 3:**
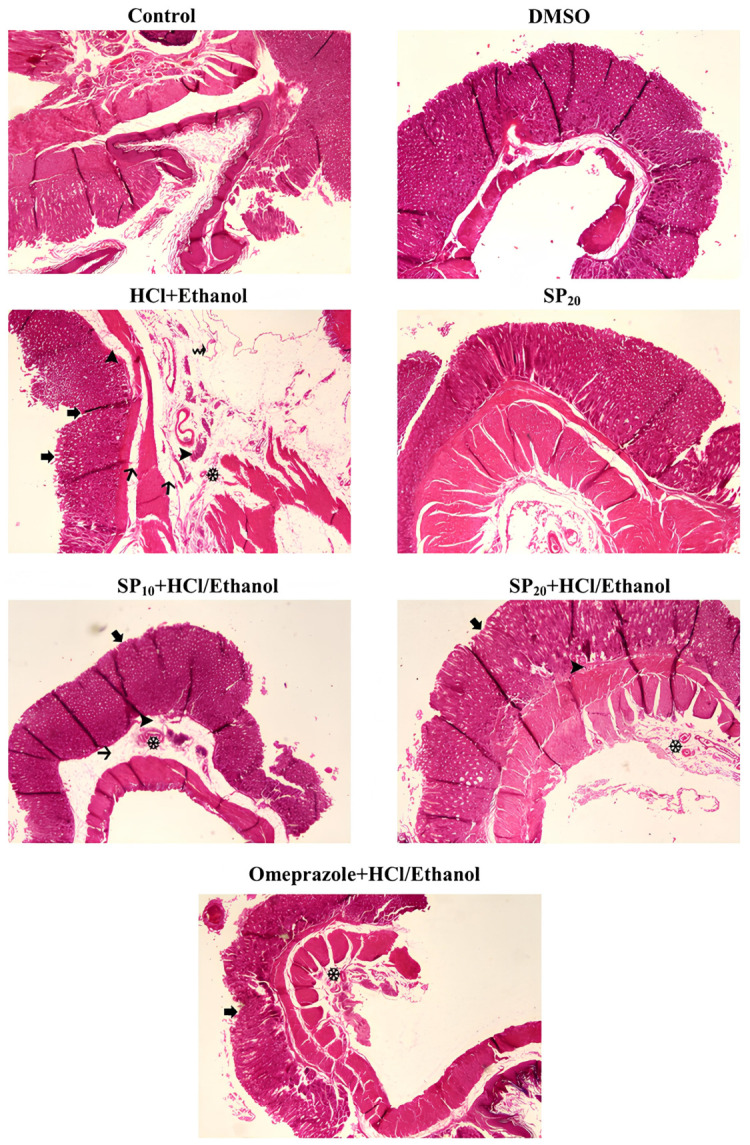
Histopathological findings in gastric tissues following acute HCl/ethanol exposure (H&E staining). Representative gastric tissue sections from the experimental groups are shown. In the HCl/ethanol-treated group, prominent epithelial desquamation and surface shedding were observed (thick arrows). Marked edema was evident within the submucosal and serosal layers (thin arrows), accompanied by mononuclear cell infiltration in these regions (arrowheads). In addition, dilation of lymphatic vessels (curved arrows) and pronounced vascular hyperemia (asterisks) were detected. Pretreatment with spinosin or omeprazole noticeably alleviated these pathological alterations, leading to improved preservation of gastric tissue architecture. Original magnification: ×100. Histopathological evaluation was performed in 7 animals per group.

**Figure 4:**
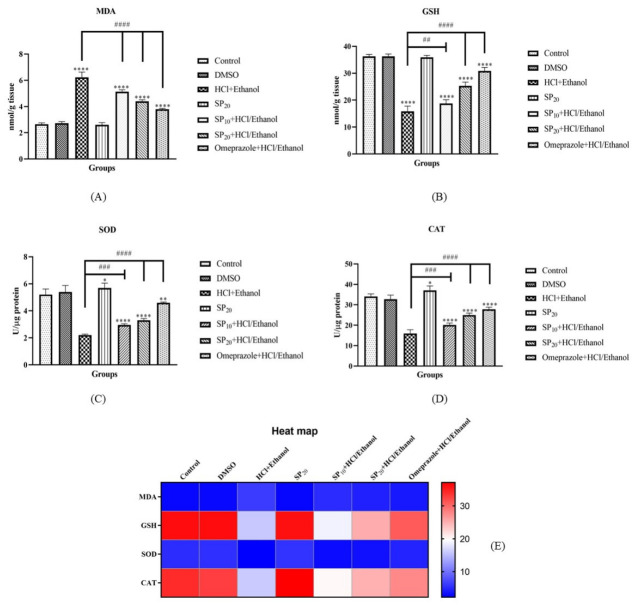
Effects of spinosin on oxidative stress markers and antioxidant defense systems in gastric tissue. Panels (**A**–**D**) illustrate the levels of malondialdehyde (MDA), reduced glutathione (GSH), superoxide dismutase (SOD), and catalase (CAT) determined in gastric tissue homogenates from the experimental groups. Acute HCl/ethanol administration resulted in a significant elevation of MDA levels, indicating enhanced lipid peroxidation, accompanied by marked reductions in GSH content and antioxidant enzyme activities (SOD and CAT) compared with the control groups. Pretreatment with spinosin (SP_10_ and SP_20_) significantly mitigated oxidative damage and restored antioxidant capacity. Comparable protective effects were observed in the omeprazole-treated group. Panel (**E**) presents a heat map summarizing the relative changes in oxidative stress and antioxidant parameters across all groups, providing an integrated visualization of the protective trends. Data are expressed as mean ± SD (n = 7 animals per group). Statistical significance is indicated as * *p* < 0.05, ** *p* < 0.01, and **** *p* < 0.0001 versus the control group; ## *p* < 0.01, ### *p* < 0.001, and #### *p* < 0.0001 versus the HCl/ethanol group.

**Figure 5:**
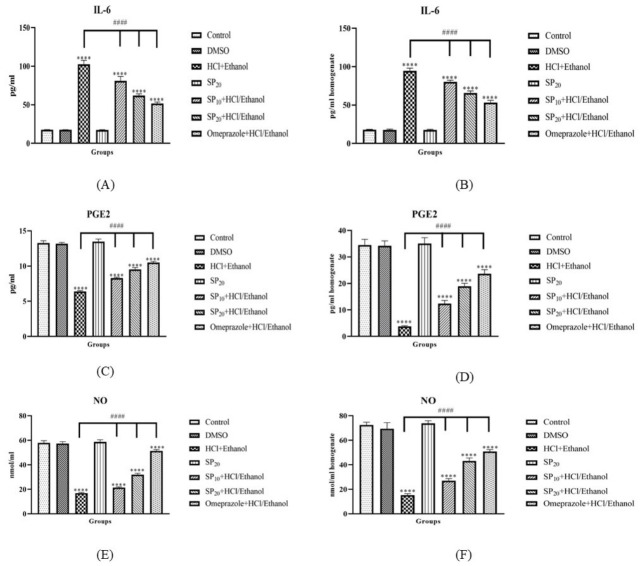
Effects of spinosin on serum and gastric tissue levels of inflammatory mediators following acute HCl/ethanol-induced injury. Panels (**A**) and (**B**) show interleukin-6 (IL-6) concentrations measured in serum and gastric tissue homogenates, respectively. Panels (**C**,**D**) present prostaglandin E_2_ (PGE_2_) levels in serum and tissue, while panels (**E**,**F**) depict nitric oxide (NO) levels determined in serum and gastric homogenates. Administration of HCl/ethanol significantly increased IL-6 levels while markedly reducing PGE_2_ and NO compared with the control groups. Pretreatment with spinosin (SP_10_ and SP_20_) significantly reversed these alterations by lowering IL-6 concentrations and restoring PGE_2_ and NO levels. Similar modulatory effects were observed in the omeprazole-treated group. Data are expressed as mean ± SD (n = 7 animals per group). Statistical significance is indicated as and **** *p* < 0.0001 versus the control group; #### *p* < 0.0001 versus the HCl/ethanol group.

**Figure 6:**
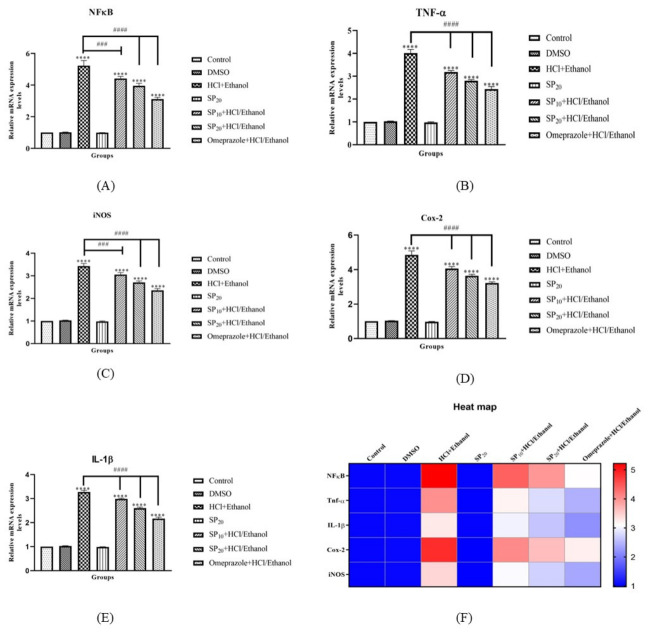
Modulatory effects of spinosin on inflammation-related gene expression in gastric tissue. Panels (**A**–**E**) illustrate the relative mRNA expression levels of nuclear factor kappa B (NFκB), tumor necrosis factor-α (TNF-α), inducible nitric oxide synthase (iNOS), cyclooxygenase-2 (Cox-2), and interleukin-1β (IL-1β) in gastric tissues from the experimental groups. Exposure to HCl/ethanol caused a significant upregulation of all examined pro-inflammatory genes compared with the control groups. Pretreatment with spinosin (SP_10_ and SP_20_) markedly attenuated the HCl/ethanol-induced increases in gene expression, demonstrating an anti-inflammatory transcriptional profile comparable to that observed with omeprazole. Panel (**F**) presents a heat map summarizing the relative expression patterns of inflammatory markers across all groups. Data are expressed as mean ± SD (n = 7 animals per group). Statistical significance is indicated as **** *p* < 0.0001 versus the control group; ### *p* < 0.001, and #### *p* < 0.0001 versus the HCl/ethanol group.

**Figure 7:**
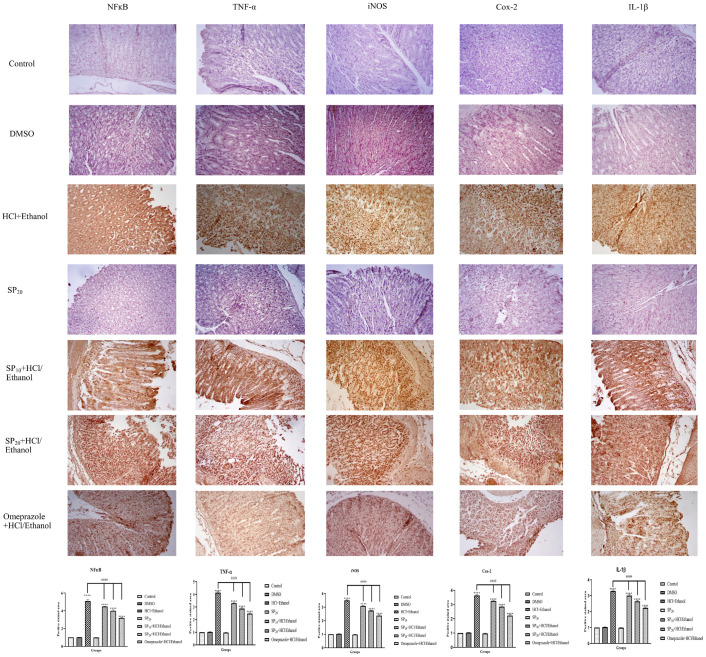
Immunohistochemical evaluation of inflammation-related markers in gastric tissue following acute HCl/ethanol-induced injury. Representative immunohistochemical staining images showing the expression patterns of nuclear factor kappa B (NFκB), tumor necrosis factor-α (TNF-α), inducible nitric oxide synthase (iNOS), cyclooxygenase-2 (Cox-2), and interleukin-1β (IL-1β) in gastric tissues from the experimental groups are presented. Minimal immunoreactivity was observed in the control and DMSO groups. In contrast, the HCl/ethanol group exhibited markedly increased immunopositive staining for all examined inflammatory markers. Pretreatment with spinosin (SP_10_ and SP_20_) substantially reduced immunoreactivity for inflammation-associated markers. The omeprazole-treated group displayed a comparable attenuation of staining intensity. Quantitative analyses of immunoreactive signals are summarized in the accompanying bar graphs. Data are expressed as mean ± SD (n = 7 animals per group). Statistical significance is denoted as **** *p* < 0.0001 versus the control group; #### *p* < 0.0001 versus the HCl/ethanol group.

**Figure 8:**
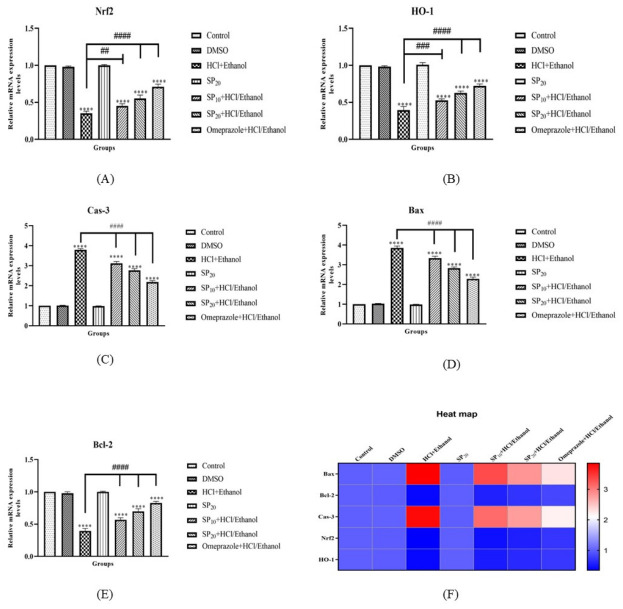
Effects of spinosin on Nrf2/HO-1 signaling and apoptosis-related gene expression in gastric tissue. Panels (**A**) and (**B**) present the relative mRNA expression levels of nuclear factor erythroid 2-related factor 2 (Nrf2) and heme oxygenase-1 (HO-1), respectively. Panels (**C**–**E**) illustrate the transcriptional expression of apoptosis-associated markers, including Cas-3 (Cas-3), Bcl-2–associated X protein (Bax), and B-cell lymphoma-2 (Bcl-2), in gastric tissues from the experimental groups. Acute HCl/ethanol exposure significantly downregulated Nrf2 and HO-1 expression while markedly upregulating pro-apoptotic markers (Cas-3 and Bax) and suppressing anti-apoptotic Bcl-2 compared with the control groups. Pretreatment with spinosin significantly reversed these alterations and was associated with increased Nrf2 and HO-1 expression together with changes in apoptosis-related gene expression. The omeprazole-treated group exhibited comparable regulatory effects. Panel (**F**) shows a heat map summarizing the relative expression patterns of oxidative stress– and apoptosis-related genes across all groups. Data are expressed as mean ± SD (n = 7 animals per group). Statistical significance is indicated as **** *p* < 0.0001 versus the control group; ## *p* < 0.01, ### *p* < 0.001, and #### *p* < 0.0001 versus the HCl/ethanol group.

**Figure 9:**
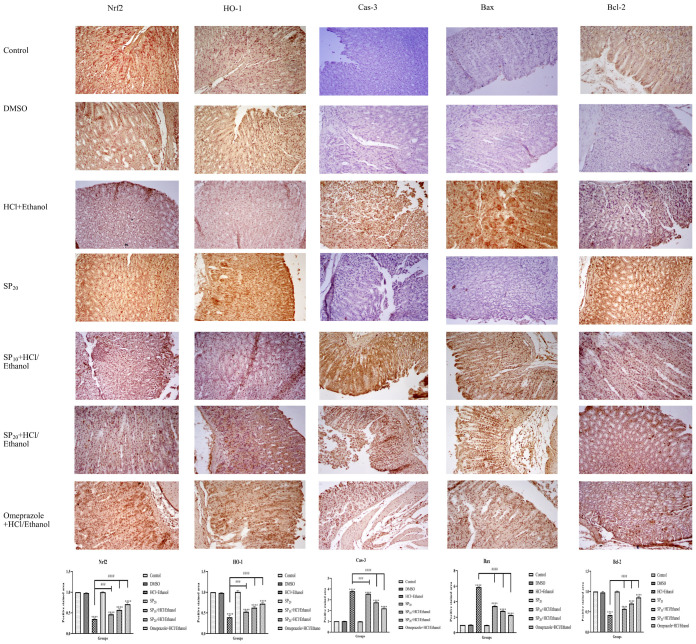
Immunohistochemical assessment of Nrf2/HO-1 and apoptosis-related markers in gastric tissue. Representative immunohistochemical images demonstrate the expression of nuclear factor erythroid 2-related factor 2 (Nrf2), heme oxygenase-1 (HO-1), caspase-3 (Cas-3), Bcl-2–associated X protein (Bax), and B-cell lymphoma-2 (Bcl-2) in gastric tissues obtained from the experimental groups. Weak basal immunoreactivity for Nrf2 and HO-1, together with the low staining intensity of apoptotic markers, was observed in the control and DMSO groups. In contrast, the HCl/ethanol group exhibited markedly reduced Nrf2 and HO-1 immunostaining accompanied by strong Cas-3 and Bax positivity and diminished Bcl-2 expression. Pretreatment with spinosin (SP_10_ and SP_20_) enhanced Nrf2 and HO-1 immunoreactivity while attenuating Cas-3 and Bax staining and restoring Bcl-2 expression, consistent with alterations in oxidative stress-related and apoptosis-associated markers. The omeprazole-treated group showed a comparable protective staining profile. Quantitative analyses of immunopositive areas are presented in the corresponding bar graphs. Data are expressed as mean ± SD (n = 7 animals per group). Statistical significance is defined as **** *p* < 0.0001 compared with the control group; ### *p* < 0.001, and #### *p* < 0.0001 compared with the HCl/ethanol group.

**Table 1:** Design of experimental groups and treatments.

Group	Treatment	Dose/Administration Route	Purpose/Description
**Control**	Physiological saline	0.1 cc/10 g, p.o.	Negative control group: received no drug treatment.
**DMSO**	0.1% DMSO	0.1 cc/10 g, p.o. (gastric gavage)	Solvent control group to assess possible DMSO effects.
**SP_20_**	Spinosin	20 mg/kg, p.o.	Received Spinosin alone to assess its baseline effect.
**HCl/Ethanol**	0.3 M HCl + 70% ethanol	0.1 cc/10 g, p.o.	Ulcer-induced group to create gastric mucosal injury.
**SP_10_ + HCl/Ethanol**	Spinosin + HCl/Ethanol	10 mg/kg Spinosin (p.o.) + 0.1 cc/10 g HCl/EtOH (p.o.)	To evaluate the gastroprotective effect of Spinosin.
**SP_20_ + HCl/Ethanol**	Spinosin + HCl/Ethanol	20 mg/kg Spinosin (p.o.) + 0.1 cc/10 g HCl/EtOH (p.o.)	To evaluate the gastroprotective effect of Spinosin.
**Omeprazole + HCl/Ethanol**	Omeprazole + HCl/Ethanol	20 mg/kg Omeprazole (p.o.) + 0.1 cc/10 g HCl/EtOH (p.o.)	Reference (positive control) group: standard anti-ulcer drug.

**Table 2:** Description of polymerase chain reaction primers (β-actin, NFκB, Cox-2, iNOS, TNF-α, IL1-β, Cas-3, Bax, Bcl-2, Nrf2, and HO-1), their sequences, and primer lengths.

Gene	Primer Sequence (5′–3′)	Primer Length (nt)
**β-actin**	F: AAGGCCAACCGTGAAAAGAT R: GTGGTACGACCAGAGGCATAC	20/21
**NFκB**	F: AGGAAGGCAAAGCGAATCCA R: TCAGAACCAAGAAGGACGGC	20/20
**Cox-2**	F: TTGCTCTCCCCTCTCTACGC R: GCAGTCGTAGTTCACCAGGTT	20/21
**iNOS**	F: AACTTGTTTGCAGGCGTCAG R: CACATTGCTCAGGGATGGA	20/19
**TNF-α**	F: ATCCATCTCTTTGCGGAGGC R: GGGGGAGAGGTAGGGATGTT	20/20
**IL-1β**	F: TGCCACCTTTTGACAGTGATG R: TGATGTGCTGCTGCGAGATT	21/20
**Cas-3**	F: AGCTTGGAACGGTACGCTAA R: CCACTGACTTGCTCCCATGT	20/20
**Bax**	F: TGCTAGCAAACTGGTGCTCA R: ATGTGGGGGTCCCGAAGTA	20/19
**Bcl-2**	F: GAACTGGGGGAGGATTGTTGG R: GCATGCTGGGGCCATATAGT	20/20
**Nrf2**	F: CATGTGTGGCAGTCCATGATTT R: AGTACTGTACAGCAGGCATAC	22/21
**HO-1**	F: CCTCACAGATGGCGTCACTT R: TGGGGGCCAGTATTGCATTT	20/20

**Table 3:** Primary antibodies, dilutions, and catalog numbers used in immunohistochemical analyses.

Antibodies	Dilutions	Catalog Number
**Bax**	1:100	E-AB-13814
**Bcl-2**	1:100	E-AB-60012
**NFĸB**	1:200	E-AB-60843
**Nrf2**	1:200	E-AB-93081
**Cox-2**	1:200	E-AB-68287
**HO-1**	1:200	E-AB-66079
**iNOS**	1:250	E-AB-70051
**Cas-3**	1:100	E-AB-30004
**IL-1β**	1:100	PAA563Ra01
**TNF-α**	1:100	PAA133Ra01

**Table 4:** Histopathological alterations in gastric tissue following administration of spinosin (SP) against HCl/ethanol exposure.

Group	Desquamation and Shedding	Submucosal Edema	Mononuclear Cell Infiltration	Lymphatic Vessel Dilation	Hyperemia in the Vessels
**Control**	0 (0–0)	0 (0–0)	0 (0–0)	0 (0–0)	0 (0–0)
**DMSO**	0 (0–0)	0 (0–0)	0 (0–0)	0 (0–0)	0 (0–0)
**HCl + Ethanol**	2 (2–3)	3 (2–3)	2 (2–3)	2 (2–3)	2 (2–3)
**SP_20_**	0 (0–0)	0 (0–0)	0 (0–0)	0 (0–0)	0 (0–0)
**SP_10_ + HCl/Ethanol**	2 (1–2)	2 (1–3)	2 (1–2)	2 (1–2)	2 (1–3)
**SP_20_ + HCl/Ethanol**	1 (1–2)	2 (1–2)	1 (1–2)	2 (1–2)	2 (1–2)
**Omeprazole + HCl/Ethanol**	1 (0–1)	1 (1–2)	1 (0–1)	0 (0–1)	1 (0–2)

Data are presented as median (minimum–maximum), n = 7 animals per group. Histopathological scores were analyzed using the Kruskal–Wallis test followed by Dunn’s multiple comparisons test. Overall differences among groups were significant for all parameters (*p* < 0.0001).

## Data Availability

All data generated or analyzed during this study are included in the manuscript.
